# Verification study of free light chains assays on reagent-optimized analysers

**DOI:** 10.11613/BM.2019.030709

**Published:** 2019-10-15

**Authors:** Dragana Šegulja, Danica Matišić, Karmela Barišić, Dunja Rogić

**Affiliations:** 1 Department of Laboratory Diagnostics, University Hospital Centre Zagreb, Zagreb, Croatia; 2 Department of Medical Biochemistry and Hematology, Faculty of Pharmacy and Biochemistry, University of Zagreb, Zagreb, Croatia

**Keywords:** M components, serum free light chains, immunoassay, gammopathy, monoclonal

## Abstract

**Introduction:**

Our aim was to compare analytical specifications of two assays (monoclonal vs. polyclonal) for free light chains (FLCs) quantification optimized for two different analytical platforms, nephelometer ProSpec (Siemens, Erlangen, Germany) and turbidimetric analyser Optilite (The Binding Site, Birmingham, UK).

**Materials and methods:**

The evaluation included verification of the precision, repeatability and reproducibility, estimation of accuracy and method comparison study with 37 serum samples of haematological patients. Kappa and lambda FLC were measured in each sample by both methods and kappa/lambda ratio was calculated.

**Results:**

Results show satisfactory precision of both methods with coefficients of variation for ProSpec of CV_wr_ = 2.20% and CV_br_ = 3.44%, and for Optilite CV_wr_ = 2.82% and CV_br_ = 4.15%. Estimated bias for FLC lambda was higher on the ProSpec analyser, but bias for FLC kappa was higher on the Optilite analyser. Correlation coefficients were 0.98; P < 0.001 for FLC kappa and 0.97; P < 0.001 for FLC lambda. Considering normal/pathological FLC ratio moderate agreement within assays was detected (κ = 0.621). When the results were categorized according to criteria for progressive disease, 4/37 (0.10) cases were differently classified. Lambda FLC values by Optilite in three samples with monoclonal FLC lambda were more than twelve times higher than by ProSpec. A 25% difference in FLC ratio was detected in 16/37 (0.43) and 50% difference in 13/37 (0.35) patients.

**Conclusions:**

All manufacturers’ precision claims could not be achieved in the verification study. The comparison of results to biological variations data showed that coefficients of variations are acceptable for both assays. The assays should not be used interchangeably in haematological patients.

## Introduction

Monoclonal protein concentration, either synthesized as an intact molecule or part of the immunoglobulin molecule (free light chain (FLC); free heavy chain) is one of the criteria in diagnosis and it is crucial in monitoring plasma cell disorders (*1*, *2*).

Traditionally used methods for measuring the concentration of M-protein are nephelometry/turbidimetry and densitometer tracing. Although nephelometry and densitometer tracing are recommended methods, there is no evidence that turbidimetry is not a good alternative in the quantification of M-protein (*1*, *3*).

Nephelometric/turbidimetric tests for measuring FLCs and heavy-light chains routinely used in clinical practice during the past decade represent valuable progress in the diagnosis and monitoring of myeloma patients (*4*-*6*).

In 2014, International Myeloma Working Group (IMWG) updated the criteria for the diagnosis of multiple myeloma. The revised IMWG criteria, in addition to the classic CRAB criteria (*i.e.* C - hypercalcaemia: serum calcium > 0.25 mmol/L higher than the upper limit of normal or > 2.75 mmol/L; R - renal insufficiency: creatinine clearance < 40 mL/min or serum creatinine > 177 μmol/L; A - anaemia: haemoglobin value of > 20 g/L below the lower limit of normal, or a haemoglobin value < 100 g/L; B - bone lesions: one or more osteolytic lesions on skeletal radiography, computed tomography (CT) or positron emission tomography-computed tomography (PET-CT), included the newly defined SLiM criteria (*i.e.* S - ≥ 60% clonal bone marrow plasma cells; Li - serum FLC ratio involved/uninvolved ≥ 100; M - > 1 focal lesion (≥ 5 mm each) detected by MRI studies) (*7*). Also, quantification of FLCs is included in international uniform therapeutic response criteria in patients without measurable serum or urine M-protein (*6*).

Free light chains antibodies in reagents should react only with exposed FLC epitopes which are hidden when the light chain is bound to the heavy chain. In addition to tests included in this evaluation with polyclonal sheep and monoclonal antibodies at least four different tests, monoclonal or polyclonal origin are available (*8*). The first relevant FLCs studies were made with a polyclonal sheep test that is no longer the only one available on the market (*6*, *9*).

Although the monoclonal reagent is applicable solely on nephelometric analytical platforms, the polyclonal reagent is applicable as an open channel test on various turbidimetric analysers with no exactly defined antigen excess parameters for FLC test and with the need for additional manual dilutions (*5*).

Taking into account the three most important sources of variability and differences in the results of immunoassays (*i.e.* prozone effect, cross reactivity and matrix influence) we prepared the verification protocol on Optilite (The Binding Site, Birmingham, UK) and ProSpec (Siemens, Erlangen, Germany) analysers for FLC assays (*10*). Optilite is a new generation special protein analyser optimized for The Binding Site (TBS) reagents with reaction kinetic method for identifying antigen excess. ProSpec uses a built-in prereaction protocol to ensure detection of antigen excess (*11*). These analytical systems have different ways of detecting the resulting immune complex; Optilite by measuring turbidity and ProSpec by measuring the amount of scattered light. Also, the difference in the reagent composition of these two manufacturers is significant. The Optilite reagent is of polyclonal origin while the Siemens reagent contains monoclonal antibodies.

In tertiary care hospital with preselected haematological patients is of great interest to use the evaluated test and to detect possible discordance of results using different FLC tests. The aim is to present the verification results of two assays for FLCs quantification optimized for two analytical platforms.

## Materials and methods

### Materials

Analysers Optilite (The Binding Site, Birmingham, United Kingdom) and ProSpec (Siemens, Erlangen, Germany) were included in the verification protocol for measuring FLCs. Tests are performed according to the manufacturer`s instructions and with automated dilutions. The same reagent lot was used during evaluation. Concentration ranges of the used control materials optimized for each reagent were for Level 1 10-30 mg/L and for Level 2 30-60 mg/L. Method comparison study, approved by the hospital ethical committee, included 37 serum samples from outpatients managed at the Department of Hematology during the evaluation week. Kappa and lambda FLC were measured in each sample by both methods and FLC kappa/lambda ratio was calculated.

### Methods

The evaluation included precision verification through testing repeatability and reproducibility, estimation of accuracy and comparison of results obtained by the applied methods. Precision was investigated analysing control materials in two concentrations, in triplicates during five days following the Clinical and Laboratory Standard Institute (CLSI) guideline EP-15 A2 (*12*). Repeatability was calculated as CV_wr_(%) using daily standard deviation (S_d_) in three replicates, and average standard deviation (S_r_) in five runs._._ Reproducibility was calculated as CV_br_(%) by dividing standard deviation (S_b_)_,_ calculated from daily means, with an average of all results (grand mean) (*13*). Results of precision experiment were compared to with-in run and between run coefficients of variation obtained during manufacturer method validation and with quality goals derived from biological variation data. Optimum, desirable and minimum specifications for imprecision were calculated as follows: I_optimum_ = 0.25 x CV_within-subject_; I_desirable_ = 0.50 x CV_within-subject_; I_minimum_ = 0.75 x CV_within-subject_.

Due to the lack of certified reference material (CRM), accuracy was estimated using results from the precision experiment procedure relative to the assigned (target) values of the control materials used in the experiment. Optimum, desirable and minimum specifications for bias were calculated as follows: B_optimum_ = 0.125x (CV_within-subject_^2^ + CV_between-subject_^2^)^1/2^, B_desirable_ = 0.25 x (CV_within-subject_^2^ + CV_between-subject_^2^)^1/2^, B_minimum_ = 0.375 x (CV_within-subject_^2^ + CV_between-subject_^2^)^1/2^.

Reference ranges indicated by each company were used for categorization of results (TBS: FLC kappa 3.30-19.40 mg/L, FLC lambda 5.71-26.30 mg/L, FLC ratio 0.26-1.65; Siemens: FLC kappa 6.70-22.4 mg/L, FLC lambda 8.30-27.00 mg/L, FLC ratio 0.31-1.56).

Results are categorized also according to criteria for progressive disease where FLC kappa or FLC lambda > 100 mg/L and 0.01 > FLC kappa/lambda ratio > 100 and to 25% and 50% difference in FLC ratio (*14*).

In order to clarify the discordance in FLC lambda results in three patient samples, serum protein electrophoresis by Capillarys 2 (Sebia, Lyses, France) was performed. Immunofixation electrophoresis using the Hydrasys system (Sebia, Lyses, France) was performed as a confirmation method for M-protein detection (*1*). Immunofixation is done by free kappa (ĸf), free lambda (λf), gamma (γ), kappa (ĸ) and lambda (λ) antisera. Also, in a sample with detected monoclonal immunoglobulin G (IgG), total IgG is measured on Cobas 6000cee (Roche Diagnostics, Rotkreuz, Switzerland).

### Statistical analysis

All data sets have been tested for normality using the Kolmogorov-Smirnov test. Correlations are described by Spearman`s rank correlation coefficients and method comparison were done by Passing-Bablok regression. The level of agreement was evaluated by the weighted Kappa coefficient. The values P ≤ 0.05 were considered statistically significant. Statistical analysis was done by MedCalc statistical software version 17.2 (Ostend, Belgium).

## Results

In precision experiment, maximum coefficients of variation (CV) were of CV_wr_ = 2,20% and CV_br_ = 3.44% for ProSpec and CV_wr_ = 2.82% and CV_br_ = 4.15% for Optilite. Although the Siemens company has declared lower CVs for the used control materials (FLC kappa CV_wr_ = 1.8% and CV_br_ = 2.2%; FLC lambda CV_wr_ = 1.9% and CV_br_ = 3.1%) during method validation, we did not manage to repeat such precision in routine verification. For FLC lambda CV_wr_ and for FLC kappa CV_br_ determined on Optilite with control material Level 2 were higher than achieved during manufacturer’s validation based on document CLSI EP5-A2: FLC kappa CV_wr_ = 3.3% and CV_br_ = 3.0%; FLC lambda CV_wr_ = 2.0% and CV_br_ = 2.6%. Comparing results to biological variation data, imprecisions for FLC lambda were within the minimum goal of 3.6%, but a minimum goal for FLC kappa of 3.6% is too strict criteria for Optilite analyser at control Level 2 (Table 1) (*15*).

**Table 1:** Repeatability and reproducibility of FLC kappa and FLC lambda by both analytical systems

**Analyser**	**Level 1**	**Level 2**	**Declared CVs**	**Goals for imprecision (CV%)**		
	**Repeatability, FLC kappa (CV_wr_%)**			**Optimum**	**Desirable**	**Minimum**
ProSpec	1.93	2.02	1.8	1.2	2.4	3.6
Optilite	2.78	1.40	3.3			
	**Reproducibility, FLC kappa (CV_br_%)**					
ProSpec	2.15	2.61	2.2			
Optilite	2.31	4.15	3.0			
	**Repeatability, FLC lambda (CV_wr_%)**					
ProSpec	1.24	2.20	1.9			
Optilite	1.15	2.82	2.0			
	**Reproducibility, FLC lambda (CV_br_%)**					
ProSpec	3.44	3.23	3.1			
Optilite	1.47	1.64	2.6			
CV – coefficient of variation. CV_wr_ – repeability. CV_br_ – reproducibility. FLC – free light chain.						

Estimated bias for FLC lambda was higher for ProSpec (Level 1 = 8.3%; Level 2 = 7.3%) than for Optilite system (Level 1 = 2.6%; Level 2 = 6.5%). ProSpec FLC kappa assay showed a bias of only 0.3% at Level 1 and 1.4% at Level 2 while bias on Optilite was 5.1% and 7.3%, respectively. Except for bias for FLC kappa on ProSpec, results were not within a minimum analytical goal for bias regarding biological variation data (FLC kappa B_optimum_ 2.0%, B_desirable_ 4.0%, B_minimum_ 6.0%; FLC lambda B_optimum_ 2.2%, B_desirable_ 4.5%, B_minimum_ 6.0% (*15*).

**Figure 1:**
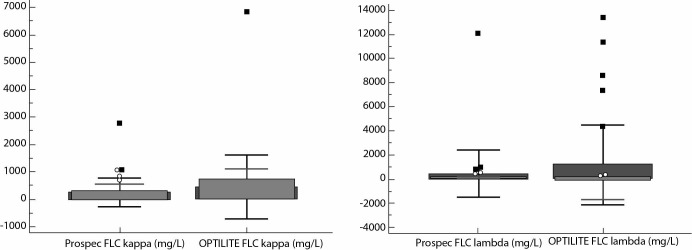
Distribution of kappa (A) and lambda (B) free light chaines (FLC) results in serum samples (N = 37) by two assays on ProSpec and Optilite analysers.

**Figure 2:**
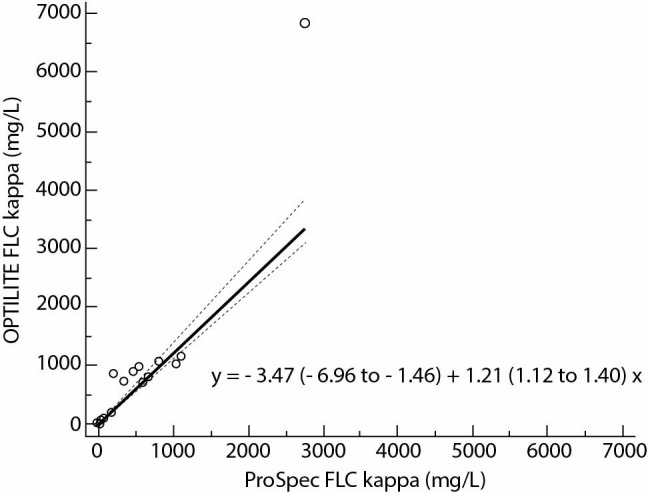
Method comparison results with 95% confidence interval (N = 37) of FLC kappa assays on ProSpec and Optilite analysers. The regression equation indicates the existence of constant and proportional error (P = 0.740). FLC - free light chain.

**Figure 3:**
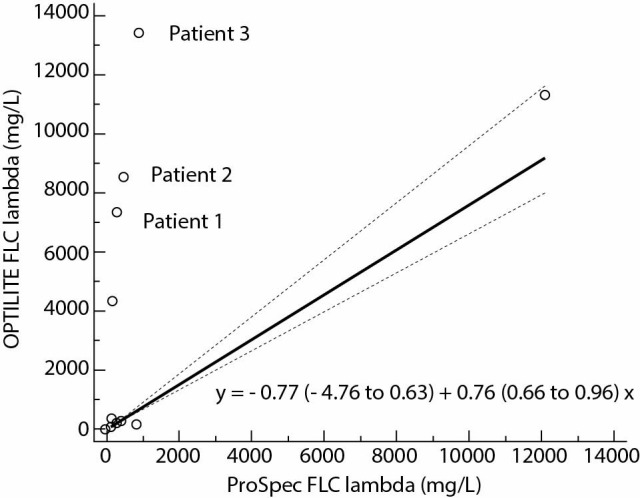
Method comparison results with 95% confidence interval (N = 37) of FLC lambda assays on ProSpec and Optilite analysers. The regression equation indicates the existence of proportional error (P = 0.460). Patients 1-3 are samples with FLC lambda values more than twelve times higher by Optilite than by ProSpec analyzer. FLC - free light chain.

Calculated Spearman`s rank correlation coefficients, r_s,_ were 0.98; P < 0.001 for FLC kappa and 0.97; P < 0.001 for FLC lambda. The regression analysis included wide concentration range (Figure 1, Table 2) and pointed to the existence of constant and proportional error in assays for FLC kappa and proportional error in FLC lambda assays: y = - 3.47 (- 6.96 to - 1.46) + 1.21 (1.12 to 1.40) x with P = 0.740; FLC lambda, y = - 0.77 (- 4.76 to 0.63) + 0.76 (0.66 to 0.96) x, with P = 0.460 (Figures 2 and 3).

**Table 2:** Concentrations of FLC kappa and FLC lambda with FLC ratios

**FLC kappa (mg/L)**	**Range**	**Median**	**IQR**
ProSpec	6.15-2770.00	24.05	16.80-279.50
Optilite	5.58-6800.14	24.48	15.35-721.29
**FLC lambda (mg/L)**			
ProSpec	1.11-12100.40	30.80	13.87-166.25
Optilite	1.37-13380.22	21.49	7.93-108.71
**FLC ratio**			
ProSpec	0.002-432.432	1.243	0.533-3.244
Optilite	0.001-654.029	1.479	0.704-4.596
FLC – free light chain. IQR – interquartile range.			

**Figure 4:**
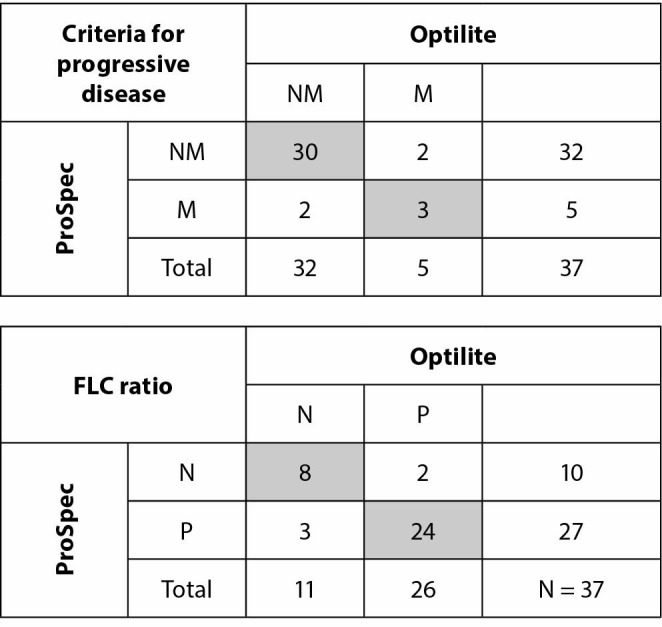
Concordance analysis according to criteria for progressive disease (FLC kappa or FLC lambda > 100 mg/L; 0.01 > kappa/lambda ratio > 100) and to FLC ratio. Reference ranges for FLC ratio were defined by the manufacturer. FLC – free light chains. M - meets criteria. NM - does not meet the criteria. N - within reference range. P - outside reference range. The grey area indicates the number of samples that gave concordant results.

If results were categorized by FLC kappa/lambda ratio, 5/37 (0.14) patients would be differently categorized considering normal or pathological ratio using these two assays. This moderate agreement of weighted Kappa 0.62 is statistically significant, P = 0.005. Furthermore, when the results are categorized according to criteria for progressive disease (FLC kappa or FLC lambda > 100 mg/L; 0.01 > kappa/lambda ratio > 100) in 2/37 (0.05) cases progressive disease would not be recognized (weighted Kappa 0.72, P = 0.003) when using one of the applied analysers (Figure 4).

The difference in FLC kappa/lambda ratios between evaluated assays was greater than 25% in 16/37 (0.43) patients and even equal to or greater than 50% in 13/37 (0.35) patients.

The method comparison graph has revealed three samples with a difference in FLC lambda results greater than twelve times (Figure 3). In these samples, the FLC lambda obtained on ProSpec was < 1000 mg/L and on Optilite > 7000 mg/L; the monoclonal synthesis of FLC lambda was confirmed by immunofixation electrophoresis (Table 3).

**Table 3:** Results of serum protein electrophoresis and immunofixation

	**Patient 1**		**Patient 2**		**Patient 3**	
FLC kappa (mg/L) (ProSpec/Optilite)	31.9/55.3		5.7/14.8		22.0/24.2	
FLC lambda (mg/L) (ProSpec/Optilite)	302.0/7313.1		541.0/8522.0		959.0/13380.2	
FLC ratio (ProSpec/Optilite)	0.11/< 0.01		0.01/< 0.01		0.02/< 0.01	
Electrophoresis	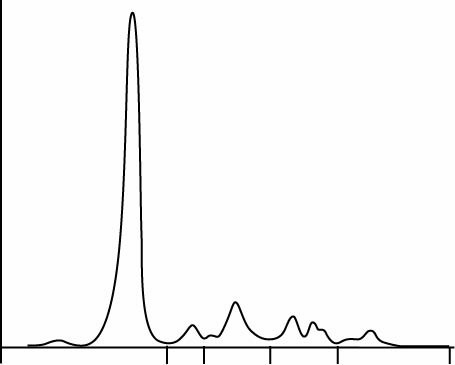		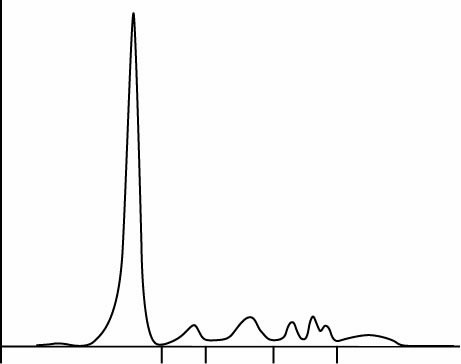		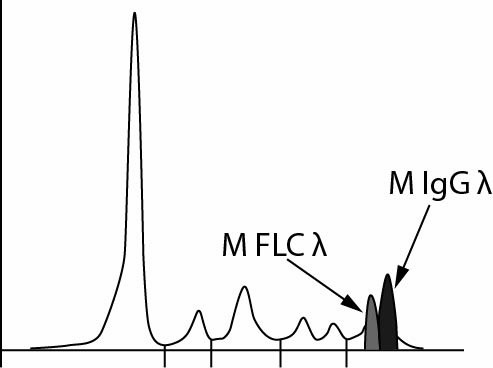	
Immunofixation electrophoresis	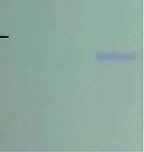 ĸf λf	Monoclonal free lambda chains	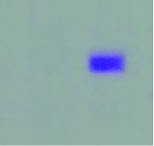 ĸf λf	Monoclonal free lambda chains	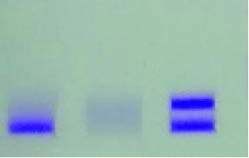 IgG ĸ λ	Monoclonal IgG lambda + monoclonal free lambda chains
FLC – free light chain. Immunofixation is done by free kappa (ĸf), free lambda (λf), gamma (γ), kappa (ĸ) and lambda (λ) antisera. In all three samples is detected M-protein FLC lambda type. In Patient 3 total IgG on Cobas 6000cee (Roche Diagnostics, Rotkreuz, Switzerland) was 10.8 g/L and by densitometric analysis IgG λ monoclonal component was 5.2 g/L and monoclonal FLC λ 3.4 g/L.						

Patients 1 and 2 were multiple myeloma patients who at the time of sampling were on therapy regimen. Four years ago, Patient 1 was monitored by nephrologist due to progressive renal disease but until sampling time he is without the need for permanent haemodialysis. At the time of sampling, he was on VMP therapy (bortezomib, melphalan, prednisone). Patient 2 was in relapse and on therapy by CyBorDex (cyclophosphamide, bortezomib and dexamethasone) protocol. Patient 3 arrived from a general hospital for the planned autologous transplantation of peripheral blood stem cells within the treatment of multiple myeloma IgG kappa. Last results in general hospital laboratory with monoclonal reagent were: FLC kappa 2310.0 mg/L, FLC lambda 19.2 mg/L, FLC ratio 0.01.

## Discussion

Our results indicate satisfactory precision between assays of the monoclonal *vs.* polyclonal origin and are comparable to recently published results authors White-Al Habeed NMA *et al.* (*16*). Regression analysis indicates the existence of proportional error in results. The difference observed in FLC lambda results in three multiple myeloma patients should not be overlooked. Similar discrepancies between two tests have also been described in previous evaluations (*17*, *18*). Messiaen AS *et al*. documented a lower correlation coefficient for FLC lambda (0.93) than FLC kappa (0.98) results in comparison of monoclonal and polyclonal reagent on the same analyser (*19*). Our results demonstrate that occasionally progressive disease would not be recognized in certain patients using one of the applied analysers. In two of three patients with monoclonal FLC lambda, during the period of active disease monitoring, the difference in FLC ratios could lead to the different interpretation of disease progression. That is especially important for Patient 3 who has been previously monitored in a different hospital. As an additional contribution to increasing FLCs values in Patient 1 can be a renal failure from anamnestic data (*20*, *21*).

Numerous studies present evaluation results of FLCs test. Most of them involve evaluation of polyclonal reagent on different not reagent-optimized analytical platforms for FLCs tests (*19*, *22*). This evaluation study is conducted on reagent-optimized analysers. The main limitation of the study is a number of included patients. Despite the fact that recently published study included a greater number of patients, our results on group of haematological patients at the tertiary level of health care present valuable contribution to the interpretation of FLC assays results (*16*).

Overestimation of FLC can be a result of non-specific interference due to aggregation (*23*). Although we obtained extremely high results by turbidimetric assay, Di Noto *et al.* found, by using sodium dodecyl sulphate-polyacrylamide gel electrophoresis (SDS-Page), dimers in all samples with significant differences between the two nephelometric FLC assays and confirmed the hypothesis that shape, size and amounts of epitopes in macromolecular complexes lead to different light scattering (*24*). Also, the difference in epitope structure due to polymerisation may lead to immunocomplexes not recognized by the monoclonal reagent. Lower results obtained by monoclonal antibodies may be the consequence of abnormal amino acid sequences or conformational changes of epitopes (*23*). Although Cigliana *et al*. suggest that internationally available standard should help to harmonise results, this would not solve test result discrepancies in certain patients (*25*).

Even though differences and possible interferences of immunoassays in general are well known, the variability of M-protein structure should be emphasized as an additional challenge in developing an immunoassay for M-protein quantification.

Changes in the plasma cell genome are numerous and substantially heterogeneous, resulting in a protein product of unpredictable structure (*5*). In lymphoproliferative diseases, changes in the immunoglobulin molecule may affect both the F_c_ and the F_ab_ domain, thus leading to the inability of using tests which recognize specific epitopes on an immunoglobulin molecule. We hypothesized that methods that include the ability to detect structure equivalence may have a certain advantage in quantifying M-protein.

From our results we can conclude that the use of different FLCs assays, even on reagent-optimized analysers, can in some patients during therapy regimen lead to different categorization of disease progression. Observed differences in clonality marker, FLC ratio represent evidence that these methods should not be used interchangeably. Furthermore, the used method for FLCs should be obligatory information on the laboratory report.

## References

[1] Dimopoulos M Kyle R Fermand JP Rajkumar SV San Miguel J Chanan-Khan A Consensus recommendations for standard investigative workup: report of the International Myeloma Workshop Consensus Panel 3. Blood. 2011;117:4701–5. 10.1182/blood-2010-10-299529 21292778

[2] Paiva B Van Dongen JJM Orfao A New criteria for response assessment: Role of minimal residual disease in bmultiple myeloma. Blood. 2015;125:3059–68. 10.1182/blood-2014-11-568907 25838346 PMC4513329

[3] Keren DF Schroeder L Challenges of measuring monoclonal proteins in serum. Clin Chem Lab Med. 2016;54:947–61. 10.1515/cclm-2015-0862 26910744

[4] Batinić J Perić Z Segulja D Last J Prijić S Dubravčić K Immunoglobulin heavy/light chain analysis enhances the detection of residual disease and monitoring of multiple myeloma patients. Croat Med J. 2015;56:263–71. 10.3325/cmj.2015.56.263 26088851 PMC4500978

[5] Jenner E Serum free light chains in clinical laboratory diagnostics. Clin Chim Acta. 2014;427:15–20. 10.1016/j.cca.2013.08.018 23999048

[6] Dispenzieri A Kyle R Merlini G Miguel JS Ludwig H Hajek R International Myeloma Working Group guidelines for serum-free light chain analysis in multiple myeloma and related disorders. Leukemia. 2009;23:215–24. 10.1038/leu.2008.307 19020545

[7] Rajkumar SV Dimopoulos MA Palumbo A Blade J Merlini G Mateos MV International Myeloma Working Group updated criteria for the diagnosis of multiple myeloma. Lancet Oncol. 2014;15:e538–48. 10.1016/S1470-2045(14)70442-5 25439696

[8] The Binding Site Group. Overview of commercial FLC assays. Available at: http://www.wikilite.com/overview-of-commercial-flc-assays. Accessed June 26th 2019.

[9] Moreau C Rouger E Henriot B Escoffre M Sebillot M Lamy T Evaluation of the concordance of two free light chains assays to identify high risk smoldering myeloma patients. Blood. 2016;128:2070 [cited 2019 June 26th] Available at http://www.bloodjournal.org/content/128/22/2070

[10] Miller JJ Levinson SS Interferences in Immunoassays. Immunoassay. 2007;25:165–90.

[11] Bossuyt X Delforge M Reynders M Dillaerts D Sprangers B Fostier K Antigen excess detection by automated assays for free light chains. Clin Chem Lab Med. 2018;56:e235–8. 10.1515/cclm-2017-0977 29679525

[12] Clinical and Laboratory Standards Institute (CLSI). User verification of performance for precision and trueness; approved guideline – second edition. CLSI document EP15-A2. Wayne, PA, USA: CLSI; 2008.

[13] Chesher D Evaluating assay precision. Clin Biochem Rev. 2008;29 Suppl 1:S23–6. 18852851 PMC2556577

[14] Kyle RA Rajkumar SV Criteria for diagnosis, staging, risk stratification and response assessment of multiple myeloma. Leukemia. 2009;23:3–9. 10.1038/leu.2008.291 18971951 PMC2627786

[15] European federation of clinical chemistry and laboratory medicine (EFLM). EFLM Biological Variation Database. Available at: https://biologicalvariation.eu/. Accessed: June 26th 2019.

[16] White-Al Habeeb NMA Earle T Spencer M Blasutig IM Evaluation of the N-latex serum free light chain assay on the Siemens BNII analyzer and agreement with The Binding Site FreeLite assay on the SPAPlus. Clin Biochem. 2018;51:90–6. 10.1016/j.clinbiochem.2017.05.009 28512013

[17] Maisin D Lepoutre T Gruson D Wallemacq P Quantification of serum free light chain kappa and lambda by the SPAPLUSanalyser. Clin Biochem. 2013;46:622–6. 10.1016/j.clinbiochem.2012.12.015 23291296

[18] Sabatino R Perrone A Cuomo M Liotti S Barchiesi V Cantile M Analytical criticalities associated to different immunological methods for serum free light chain detection in plasma cell dyscrasias: A description of particular clinical cases. Int J Mol Sci. 2017;18:804. 10.3390/ijms18040804 28417905 PMC5412388

[19] Messiaen AS De Sloovere MMW Claus PE Vercammen M Van Hoovels L Heylen O Performance Evaluation of Serum Free Light Chain Analysis: Nephelometry vs Turbidimetry, Monoclonal vs Polyclonal Reagents. Am J Clin Pathol. 2017;147:611–22. 10.1093/ajcp/aqx037 28575180

[20] Hutchison CA Harding S Hewins P Mead GP Townsend J Bradwell AR Quantitative assessment of serum and urinary Polyclonal free light chains in patients with chronic kidney disease. Clin J Am Soc Nephrol. 2008;3:1684–90. 10.2215/CJN.02290508 18945993 PMC2572283

[21] Hutchison CA Plant T Drayson M Cockwell P Kountouri M Basnayake K Serum free light chain measurement aids the diagnosis of myeloma in patients with severe renal failure. BMC Nephrol. 2008;9:11. 10.1186/1471-2369-9-11 18808676 PMC2564915

[22] Cotten SW Shajani-Yi Z Cervinski MA Voorhees T Tuchman SA Korpi-Steiner N Reference intervals and diagnostic ranges for serum free κ and free λ immunoglobulin light chains vary by instrument platform: Implications for classification of patient results in a multi-center study. Clin Biochem. 2018;58:100–7. 10.1016/j.clinbiochem.2018.06.003 29885308

[23] Tate J Bazeley S Sykes S Mollee P Quantitative serum free light chain assay - analytical issues. Clin Biochem Rev. 2009;30:131–40. 19841696 PMC2755002

[24] Di Noto G Cimpoies E Dossi A Paolini L Radeghieri A Caimi L Polyclonal versus monoclonal immunoglobulin-free light chains quantification. Ann Clin Biochem. 2015;52:327–36. 10.1177/0004563214553808 25225181

[25] Cigliana G Gulli F Napodano C Pocino K De Santis E Colacicco L Serum free light chain quantitative assays: Dilemma of a biomarker. J Clin Lab Anal. 2018;32:e22243. 10.1002/jcla.22243 28444965 PMC6817218

